# Critical Care Nurses' Perception of Medication Administration Errors in Kelantan, Malaysia: A Cross-Sectional Study

**DOI:** 10.1155/2024/3055826

**Published:** 2024-03-31

**Authors:** Muhammad Solehuddin Ishak, Mohd Ismail Ibrahim

**Affiliations:** Department of Community Medicine, School of Medical Sciences, Universiti Sains Malaysia, Kubang Kerian 16150, Kota Bharu, Kelantan, Malaysia

## Abstract

**Background:**

The study investigates critical care nurses' perceptions of medication administration errors (MAEs) in a tertiary hospital in Kelantan, Malaysia, within the unique sociocultural context of East-Coast Malaysia. The research aims to understand the causes and underreporting of MAEs and assess the proportion of reported incidents according to MAE types.

**Methods:**

A cross-sectional study involving 424 critical care nurses from Hospital Raja Perempuan Zainab II (HRPZII), Hospital Sultan Ismail Petra (HSIP), and Hospital Tanah Merah (HTM) was conducted. Nurses in administrative roles or unavailable during the survey period were excluded. The study utilized a validated Medication Administration Errors Survey questionnaire to gauge nurses' perceptions on the causes of MAEs, reasons for underreporting, and the percentage of reported incidents based on MAE types.

**Results:**

Results indicate that illegible medication orders from doctors were identified as the primary cause of MAEs, while a lack of 24-hour pharmacist availability received the lowest score. The most common reason for not reporting MAEs was identified as the nursing administration's focus on individuals rather than systemic issues when errors occur. The majority of MAEs were non-intravenous, with incorrect timing of administration being the leading cause.

**Conclusion:**

The study sheds light on critical care nurses' perspectives on MAEs in a Malaysian hospital setting, highlighting key factors contributing to these errors and barriers to their reporting. Understanding these perceptions is crucial for developing strategies to mitigate MAEs and enhance patient safety in critical care environments.

## 1. Background

Medication administration errors (MAEs) are defined as “a deviation from the prescriber's medication order as written on the patient's chart, manufacturers' preparation/administration instructions, or relevant institutional policies” [1]. MAEs are a major concern in the nursing sector, resulting from inconsistencies between the medication delivered to patients and the intended medical therapy prescribed by healthcare practitioners [2]. In the United States alone, drug-related incidents kill between 7,000 and 9,000 people each year [3]. Medication errors can have serious consequences, such as increased patient mortality, extended hospitalization times, and increased medical costs [4–6]. Medication errors are predicted to cost the world USD 42 billion per year [7].

The major reason for reporting such errors was concern about patient safety [8]. The goals of World Patient Safety Day are to educate the public, motivate action, and encourage governments to prioritize patient safety in healthcare systems worldwide. On September 17, 2022, World Patient Safety Day was honored with the theme “Medication Safety,” highlighting the necessity of ensuring that medications are safe for patients to use [9]. The program aimed to eliminate unnecessary adverse drug reactions by consolidating the World Health Organization's Global Patient Safety Challenge: Medication without Harm [10].

The five “rights” linked with the safe and effective administration of medicines are the “right patient,” “right medication,” “right time,” “right dose,” and “right route.” Errors when delivering medications to patients often violate one or more of these rights [11]. The five “rights” have been firmly established in nursing education as standard guidelines for ensuring safe and effective medication delivery. However, new study has highlighted the concept that medication administration is merely one component of a larger and more complex pharmaceutical utilization process [3, 12]. As a result, four new “rights” were proposed: right documentation, right action/reason, right form, and right reaction [13]. Medication errors are more common in high-volume care settings including emergency rooms and intensive care units [14].

The fear of experiencing negative consequences associated with reporting and undergoing disciplinary actions, the fear of being held accountable, the fear of the response from nurse management and colleagues, and the fear of job loss all influence nurses' attitudes toward reporting medication errors [15]. The act of reporting medication errors is critical to properly resolving these situations because it facilitates knowledge learning among healthcare personnel and raises medication safety awareness [16]. Multiple factors, including insufficient professional experience, participation in night shifts, poor on-the-job training, the absence of preestablished protocols for medicine administration, and interruptions during administration procedures, have shown statistically significant relationships with medication administration errors [17]. Medication errors are more likely in the context of intravenous therapy [18].

Nurses spend 40% of their time on medication administration, making them crucial in the healthcare system as the last checkpoint before giving medications. Nurses need to detect and correct errors in keeping with their professional, legal, and ethical responsibilities [19]. The ongoing concern about medical delivery errors remains a significant feature in the goal of patient safety [13]. Identifying a persistent root cause of errors and subsequently devising a good solution that significantly minimizes the likelihood of their recurrence is a substantial and tough challenge [3].

Nurses play an important role in facilitating medication administration to patients in the hospital setting [20]. The study setting still relies on conventional paper-based methods for prescription of the medications. Nurses in critical care units at a tertiary hospital in East-Coast Malaysia are likely to speak Malay at home and at work [21]; this study aims to learn their perspectives on the factors that lead to MAEs and why some of them choose not to report them. In order to fulfil healthcare needs, Malaysia uses both locally branded medications and medications imported from other nations, such as Pfizer, as per the National Pharmaceutical Regulatory Agency (NPRA) [22]. Because of the scarcity of local study on MAEs among nurses, the current study takes advantage of this information gap. As a result, the study's objectives are to assess critical care nurses' perceptions of the causes of MAEs and the reasons for not reporting MAEs, as well as the percentage of reported non-intravenous and intravenous-related MAEs.

## 2. Materials and Methods

### 2.1. Study Design and Participants

Over a period of two months beginning in February 2023, a cross-sectional survey was undertaken among critical care nurses registered and employed at tertiary institutions in Kelantan, Malaysia. The study involved three tertiary hospitals: Hospital Raja Perempuan Zainab 2 (HRPZII), Hospital Sultan Ismail Petra (HSIP), and Hospital Tanah Merah (HTM).

The sample size was calculated using a single mean calculation according to study conducted by You et al. [23]. The lists of critical care nurses were obtained from the nursing units of each hospital. The participants were chosen at random in proportion to the quantity of nurses available during the study. There were 424 critical care nurses in all, including 276 from HRPZII, 121 from HSIP, and 27 from HTM. The inclusion criteria for this study consisted of registered nurses who were directly involved in providing patient care and had a minimum employment duration of six months at each unit. Additionally, in order to keep our emphasis on evaluating staff who interact directly with patient care, we purposefully eliminated administrative roles, nurses who were not present during the survey, and nurses who did not provide direct patient care from our consideration.

### 2.2. Measuring Tool

The Medication Administration Errors Survey, a self-reported questionnaire created by Wakefield et al., was used to assess nurses' perceptions of MAEs [24]. The questionnaire was approved for use prior to the study's conduct. It was confirmed, and Cronbach's alpha value was 0.78. The surveys were divided into three sections: 28 questions on the causes of MAEs; 16 items on why MAEs are not reported; and 20 items on the reported non-intravenous (Brabcová et al.) and IV-related MAEs (9 and 11 items, respectively). Each question in the first two parts was scored on a 6-point Likert scale (1 = strongly disagree and 6 = strongly agree). The result was presented as mean and standard deviation for each item. The third domain was presented as frequency and percentage of type of MAEs, which include non-intravenous and intravenous-related MAEs. They used a 10-point Likert scale. The participants required 10 to 20 minutes to complete the survey.

### 2.3. Data Collection

The permission to conduct the study was granted by the State Health Director and the director of each of the hospitals. The data collecting method was facilitated by the nursing units. The survey was in English format, but the participants were able to comprehend and respond appropriately. We made sure to get the informed consent of those who were chosen and willing to participate. The questionnaires were collected as soon as the participants completed the survey. Their decisions were not affected by the superior since the procedure occurred in an isolated hall for each hospital involved.

### 2.4. Data Analysis

The data were entered and analyzed using SPSS ver. 26. Descriptive statistics were used to summarize the result. The mean and standard deviation were used to portray numerical data, considering their normal distribution. Categorical data were reported as frequency and percentage. Multiple logistic regression was used to analyze the variables of age, gender, education level, and job duration, as detailed in the following.

### 2.5. Ethical Consideration

Ethical approval was obtained from the relevant committees, including Jawatankuasa Etika Penyelidikan Manusia Universiti Sains Malaysia (JEPeM-USM) dated 28th December 2022, bearing the JEPeM Code: USM/JEPeM/22110733, and the National Medical Research Register (NMRR) dated 13th January 2023, with the reference number NMRR ID-22-02882-R29. Strict confidentiality measures were adhered to, and the data analysis and reporting were conducted without revealing participants' identities.

## 3. Results

### 3.1. Sociodemographic Characteristics and Job Experience of the Participants

The study involved 424 critical care nurses.  Table 1 summarizes the sociodemographic characteristics and job characteristics of the participants in a comparable study by You et al. [23] and Bifftu et al. [25]. The mean age was 40.90 ± 6.13 years. Most of the participants are married. The mean work time was 16.83 ± 5.90 years. Most of them worked at Intensive Care Unit (ICU; 39.2%), followed by Neonatal Intensive Care Unit (NICU; 16.3%), Operation Theatre (OT; 15.3%), Emergency Department (ED; 8.3%), Cardiac Care Unit (CCU; 8.0%), High Dependency Ward (HDW; 7.3%), and Paediatric Intensive Care Unit (PICU; 5.7%). Majority of them (79.5%) worked at least 50 hours per week. In their clinical context, 84.9% of the participants reported a nurse-patient ratio of 1 : 1 to 1 : 6. Patient safety training was attended by 61.3% of participants. 86.8% of participants were instructed how to administer medications. In contrast, 84.7% and 65.6% of people, respectively, had no prior experience with MAEs, either directly or via observation. The outcome under consideration is critical care nurses' readiness to report MAEs to authorities. Most nurses (60.1%) did not believe errors should be revealed.

### 3.2. Critical Care Nurses' Perception regarding the Causes of MAEs

Table 2 shows the perception of MAE causes by the critical care nurses. According to the finding, illegible medicine orders from physicians scored the highest items (4.39 ± 1.35) followed by look-alike drugs (4.38 ± 1.47), and package similarity (4.31 ± 1.46) was the third most recognized cause of MAEs. The least cause of MAEs was pharmacists being unavailable for over 24 hours, with a mean value of (1.94 ± 1.20). The remaining items' score ranged from 2.29 to 4.27.

### 3.3. Critical Care Nurses' Perception of Reasons for Not Reporting MAEs

Table 3 indicates critical care nurses' perceptions of reasons for not reporting MAEs. The highest mean score for a reason not to report MAEs was when MAEs occur, nursing administration focuses on the individual rather than looking at the systems as a potential cause of the error (3.80 ± 1.64). The second higher mean score was when nurses could be blamed if something happens to the patient as a result of the medication error (3.69 ± 1.70), and the third reason was when the patient or family might develop a negative attitude toward the nurse or may sue the nurse if a medication error is reported (3.06 ± 1.60).

### 3.4. Perceived Reported Medication Administration Errors (MAEs) by Intravenous and Non-Intravenous (Non-IV) Routes among Critical Care Nurses

Table 4 shows the percentage of perceived MAEs for each item in Kelantan government hospitals with specialists. The data show that the MAEs with the greatest perceived intravenous drug error rates were incorrect timing (16.5%), wrong dosage (16.3%), and wrong route (16.1%). The least perceived medication error was IV fluid mismatch (14.9%). The highest percentages for non-intravenous MAEs were 18.9% for medication given at the inappropriate time, 18.6% for method, and 18.2% for dosage.

## 4. Discussion

MAE prevention is a main focus of hospital quality improvement and risk management initiatives. Medication administration is generally entrusted to nurses and is an important component of nursing practice, having a substantial impact on patient safety and the provision of high-quality healthcare services. Nurses receive medication administration training in order to reduce drug-related incidents in hospital settings, as they play a critical role in preventing such errors prior to medication administration to patients.

According to the findings of the current study, 61.3% of participants had attended patient safety courses, showing that they had received enough training on safe and effective patient care. In comparison to a survey conducted in Seoul, just 47.8% of participants had received patient safety education [26]. However, in an Ethiopian study, 84.5% of participants did not get patient safety training [27]. This is concerning because healthcare providers require adequate patient safety training to avoid making errors that endanger patients. It emphasizes the need of healthcare organizations stressing patient safety education and training for their employees [28]. Medication administration instructions were given to more than 80% of study participants. This demonstrates the importance of critical care hospitals prioritizing employee training on medication administration practices. But, only 55.7% of Ethiopian study participants obtained medical administration instruction [6].

The present study's results indicated that 84.7% of critical care nurses had no MAE experience, compared to a previous study in Turkey, where 73.9% of nurses had no MAE experience, showing that the current study had improved better in terms of practicing patient safety culture [29, 30]. However, prior MAE experience was reported by 69.6% of nurses in South Korea, demonstrating that MAE prevalence differs among healthcare systems and settings [31]. These disparities in prevalence could be attributed by differences in reporting standards or medication administration practices [32]. These findings emphasize the significance of continuing education for healthcare personnel especially critical care nurses in ensuring patient safety. According to the results of this survey, 65.6% of respondents have seen MAEs performed by others. This shows that MAEs are common in clinical settings and can risk patient safety. According to a recent Turkish study, 55.8% of respondents reported witnessing MAEs [29]. It emphasizes the significance of encouraging nurses to report and treat MAEs to improve patient safety. Having healthcare personnel report MAEs helps organizations discover areas for improvement and take preventive measures.

MAEs are causing worry in healthcare settings since they may endanger patients. MAEs can arise as a result of problems with drug ordering, dispensing, administration, and monitoring. According to critical care nurses, unclear physician prescription instructions induced MAEs. A similar finding was found in a South Korean study [31], where nurses assessed the unreadable physician's medication order as extremely crucial in contributing to MAEs. Both studies emphasize the need of legible medication instructions in avoiding MAEs. Clear and easy-to-read prescription instructions are important to avoid medication errors, and this can be achieved through standardized templates, electronic prescribing, or better physician training [33]. According to a South African study, MAEs may be caused by illegible handwriting. Prescription interpretation was performed by pharmacists 75% of the time and nurses 81.8% of the time [34]. This study highlights the significance of healthcare providers giving legible and explicit prescription instructions during medication administration [12]. The introduction of comparable drugs was the second most common cause of MAEs. It is worth noting that nurses in the United States reported this difficulty more frequently, implying that it is a more general issue [35]. When many prescriptions have identical colors or formats, it can be confusing. Critical care nurses identified this issue as the second most common cause of MAEs in this study, emphasizing its importance in medication use. Drug packaging, labelling requirements, and cultural variances may all contribute to this impression mismatch [3, 36, 37].

Nursing administration attributes prescription errors to individual acts or oversights rather than systemic issues. According to the majority of critical care nurses, nursing administration prioritized the individual over the system, which is consistent with previous study and conclusions [3, 31, 35]. Nursing administration, according to these studies, blames nurses for prescription errors rather than addressing systemic issues. This limited view of human responsibility may make it more difficult to explain drug mishaps. As a result, addressing the contextual factors driving medication errors requires a thorough strategy. Healthcare medicine administration must change for the sake of safety and efficiency [38, 39]. Another MAE myth that critical care nurses in this study perceived was that nurses may be held accountable for patient damage caused by prescription errors. According to a Saudi Arabian study, nurses were unwilling to report medication mistakes for fear of being implicated [40]. According to this study, nurses may be hesitant to disclose prescription errors for fear of disciplinary or legal penalties. This emphasizes the need of building a safety culture in healthcare companies that encourages reporting and learning from errors rather than condemning nurses [41]. Such an approach may improve patient safety and service quality by encouraging openness and responsibility [41, 42].

In this study, non-intravenous and intravenous MAEs included medication administration at the inappropriate time, route, and dose. In non-IV and IV-related MAEs, medication was rarely given without a doctor's order. Medication distribution at an inconvenient time was the most common IV-related MAE in Saudi Arabia [40]. South Korean studies produced a range of results. Giving medicine to the wrong patient was the most common non-intravenous MAE, while improper drug infusion rate was the most common IV-related MAE [31]. Effective communication among the healthcare team and adherence to medication order protocols may help decrease MAEs. Ongoing education, training, and monitoring are essential for ensuring medication safety [6].

This study does have a few limitations. It is based on data that participants reported themselves, which could be influenced by biases. These biases could include misremembering past events, wanting to appear in a positive light, and interpreting questions differently. Most of the people who took part in the study are critical care nurses who are 40 years old. It is possible that the responses are biased because people who have made or seen medication errors might be more willing to admit them. Using questionnaires to collect self-reported data can limit the amount of detailed information about how often medication errors happen or how serious they are.

## 5. Conclusion

In conclusion, the study looked into the perceptions of critical care nurses on the causes of MAEs in public hospitals with specialists in Kelantan, as well as the reasons for not reporting these MAEs. According to the findings of this study, illegible medication orders from physicians, drug similarity, and medication package similarity are the top contributors to MAEs in the eyes of nurses. In contrast, the key reasons why MAEs are not reported are that nursing administration focuses on persons rather than systems, nurses may be held accountable, and patients and their families may file legal action against them. The findings highlight the importance of developing comprehensive strategies to address the highlighted reasons, improve communication and training, decrease strain and time pressure, and create a safety culture that fosters error reporting and learning. Further study is required to investigate the efficacy of these approaches in reducing MAEs.

## Figures and Tables

**Table 1:** Sociodemographic characteristics of the participants (*n* = 424).

Variables	*n* (%)	Mean ± SD
Age (years)		40.90 ± 6.13

*Sex*		
Female	414 (97.6)	
Male	10 (2.4)	

*Marital status*		
Single	10 (2.4)	
Married	387 (91.3)	
Divorced	27 (6.4)	

*Level of education*		
Diploma	403 (95.0)	
BSc	21 (5.0)	

*Place of working*		
HRPZII	276 (65.1)	
HSIP	121 (28.5)	
HTM	27 (6.4)	
Working duration (year)		16.83 ± 5.90

*Working at the current unit*		
ICU	166 (39.2)	
NICU	69 (16.3)	
PICU	24 (5.7)	
CCU	34 (8.0)	
HDW	31 (7.3)	
OT	65 (15.3)	
ED	35 (8.3)	

*Average weekly work hours*		
<50	337 (79.5)	
≥50	87 (20.5)	

*Nurse-patient ratio current unit*		
1 : 1–6	360 (84.9)	
1 : 7–10	55 (13.0)	
1 : >10	9 (2.1)	

*Do you attend any patient safety courses*		
Yes	260 (61.3)	
No	164 (38.7)	

*Have you attended any courses on medication administration guidelines*		
Yes	368 (86.8)	
No	56 (13.2)	

*Have you experienced any MAEs*		
Yes	65 (15.3)	
No	359 (84.7)	

*Have you watched any MAEs by others*		
Yes	146 (34.4)	
No	278 (65.6)	

*In your perception, when you experience or watch any MAEs, would you report the incident to the authority*		
Yes	169 (39.9)	
No	255 (60.1)	

**Table 2:** The perception of the causes of MAEs among critical care nurses in tertiary hospitals, Kelantan (*n* = 424).

Items	Strongly disagree, *n* (%)	Moderately disagree, *n* (%)	Slightly disagree, *n* (%)	Slightly agree, *n* (%)	Moderately agree, *n* (%)	Strongly agree, *n* (%)	Mean ± SD^*∗*^
(1) The names of many medications are similar	43 (10.1)	38 (9.0)	37 (8.7)	124 (29.2)	102 (24.1)	80 (18.9)	4.05 ± 1.54
(2) Different medications look alike	29 (6.8)	27 (6.4)	44 (10.4)	90 (21.2)	123 (29.0)	111 (26.2)	4.38 ± 1.47
(3) The packaging of many medications is similar	30 (7.1)	26 (6.1)	46 (10.8)	103 (24.3)	117 (27.6)	102 (24.1)	4.31 ± 1.46
(4) Physicians' medication orders are not legible	16 (3.8)	29 (6.8)	47 (11.1)	117 (27.6)	110 (25.9)	105 (24.8)	4.39 ± 1.35
(5) Physicians' medication orders are not clear	14 (3.3)	38 (9.0)	58 (13.7)	112 (26.4)	113 (26.7)	89 (21.0)	4.27 ± 1.35
(6) Physicians change orders frequently	31 (7.3)	39 (9.2)	60 (14.2)	123 (29.0)	110 (25.9)	61 (14.4)	4.00 ± 1.42
(7) Abbreviations are used instead of writing the orders out completely	44 (10.4)	54 (12.7)	69 (16.3)	113 (26.7)	82 (19.3)	62 (14.6)	3.76 ± 1.53
(8) Verbal orders are used instead of written orders	46 (10.8)	57 (13.4)	60 (14.2)	90 (21.2)	90 (21.2)	81 (19.1)	3.86 ± 1.62
(9) The pharmacy delivers incorrect doses to this unit	100 (32.6)	110 (25.9)	88 (20.8)	76 (17.9)	41 (9.9)	8 (1.9)	2.70 ± 1.37
(10) The pharmacy does not prepare the medication correctly	117 (27.6)	114 (26.9)	85 (20.0)	72 (17.0)	30 (7.1)	6 (1.4)	2.53 ± 1.32
(11) The pharmacy does not label the medication correctly	141 (33.3)	108 (25.5)	85 (20.0)	63 (14.9)	21 (5.0)	6 (1.4)	2.37 ± 1.29
(12) Pharmacists are not available 24 hours a day	211 (49.8)	105 (24.8)	59 (13.9)	27 (6.4)	17 (4.0)	5 (1.2)	1.94 ± 1.20
(13) Frequent substitution of drugs (i.e., cheaper generic for brand names)	59 (13.9)	76 (17.9)	91 (21.5)	101 (23.8)	68 (16.0)	29 (6.8)	3.31 ± 1.46
(14) Poor communication between nurses and physicians	64 (15.5)	67 (15.8)	86 (20.3)	109 (25.7)	59 (13.9)	39 (9.2)	3.35 ± 1.51
(15) Many patients are on the same or similar medications	56 (13.3)	54 (12.7)	60 (14.2)	130 (30.7)	82 (19.6)	41 (9.7)	3.60 ± 1.51
(16) Unit staff do not receive enough services on new medications	66 (15.6)	65 (15.3)	80 (18.9)	81 (19.1)	79 (18.6)	53 (12.5)	3.47 ± 1.62
(17) In this unit, there is no easy way to look up information on medications	110 (25.9)	101 (23.8)	88 (20.8)	72 (17.0)	37 (8.7)	16 (3.8)	2.70 ± 1.43
(18) Nurses in this unit have limited knowledge about medications	93 (21.9)	108 (25.5)	86 (20.3)	68 (16.0)	49 (11.6)	20 (4.7)	2.84 ± 1.47
(19) Nurses get pulled between teams and from other units	102 (24.1)	78 (18.4)	80 (18.9)	75 (17.7)	66 (15.6)	23 (5.4)	2.99 ± 1.56
(20) When scheduled medications are delayed, nurses do not communicate the time when the next dose is due	119 (28.1)	91 (21.5)	81 (19.1)	71 (16.7)	36 (8.5)	26 (6.1)	2.75 ± 1.53
(21) Nurses on this unit do not adhere to the approved medication administration procedure	167 (39.4)	100 (23.6)	68 (16.0)	56 (13.2)	20 (4.7)	13 (3.1)	2.29 ± 1.38
(22) Nurses are interrupted while administering medications to perform other duties	57 (13.4)	58 (13.7)	49 (11.6)	95 (22.4)	90 (21.2)	75 (17.7)	3.77 ± 1.66
(23) Unit staffing levels are inadequate	40 (9.4)	59 (13.9)	45 (10.6)	68 (16.0)	84 (19.8)	128 (30.2)	4.13 ± 1.71
(24) Medication orders are not transcribed to the Kardex correctly	53 (12.5)	68 (16.0)	90 (21.2)	121 (28.5)	62 (14.6)	30 (7.1)	3.38 ± 1.42
(25) Errors are made in the medication Kardex	78 (18.4)	78 (18.4)	109 (25.7)	102 (24.1)	43 (10.1)	14 (3.3)	2.99 ± 1.37
(26) Equipment malfunctions or is not set correctly (e.g., IV pump)	100 (23.6)	86 (20.3)	77 (18.2)	68 (16.0)	63 (14.9)	30 (7.1)	3.00 ± 1.60
(27) The nurse is unaware of a known allergy	107 (25.2)	100 (23.6)	86 (20.3)	92 (21.7)	28 (6.6)	11 (2.6)	2.69 ± 1.37
(28) Patients are off the ward for other care	152 (35.8)	95 (22.4)	63 (14.9)	65 (15.3)	29 (6.8)	20 (4.7)	2.49 ± 1.50

^*∗*^Min (1); max (6).

**Table 3:** The perception of the reasons for not reporting MAEs among critical care nurses in tertiary hospitals, Kelantan (*n* = 424).

Items	Strongly disagree, *n* (%)	Moderately disagree, *n* (%)	Slightly disagree, *n* (%)	Slightly agree, *n* (%)	Moderately agree, *n* (%)	Strongly agree, *n* (%)	Mean ± SD^*∗*^
(1) Nurses do not agree with the hospital's definition of a medication error	109 (25.7)	88 (20.8)	106 (25.0)	80 (18.9)	27 (6.4)	14 (3.3)	2.69 ± 1.37
(2) Nurses do not recognize an error occurred	134 (31.6)	87 (20.5)	80 (18.9)	77 (18.2)	33 (7.8)	13 (3.1)	2.59 ± 1.44
(3) Filling out an incident report for a medication error takes too much time	126 (29.7)	90 (21.2)	87 (20.5)	72 (17.0)	35 (8.3)	14 (3.3)	2.63 ± 1.44
(4) Contacting the physician about a medication error takes too much time	154 (36.3)	104 (24.5)	80 (18.9)	55 (13.0)	20 (4.7)	11 (2.6)	2.33 ± 1.34
(5) Medication error is not clearly defined	111 (26.2)	86 (20.3)	97 (22.9)	83 (19.6)	34 (8.0)	13 (3.1)	2.72 ± 1.40
(6) Nurses may not think the error is important enough to be reported	256 (60.4)	72 (17.0)	54 (12.7)	26 (6.1)	10 (2.4)	6 (1.4)	1.77 ± 1.17
(7) Nurses believe that other nurses will think they are incompetent if they make medication errors	161 (38.0)	100 (23.6)	64 (13.4)	57 (13.4)	29 (6.8)	13 (3.1)	2.37 ± 1.43
(8) The patient or family might develop a negative attitude toward the nurse or may sue the nurse if a medication error is reported	97 (22.9)	76 (17.9)	81 (19.1)	82 (19.3)	51 (12.0)	37 (8.7)	3.06 ± 1.60
(9) The expectation that medications be given exactly as ordered is unrealistic	208 (49.1)	87 (20.5)	69 (16.3)	38 (9.0)	18 (4.2)	4 (0.9)	2.02 ± 1.24
(10) Nurses are afraid the physician will reprimand them for the medication error	165 (38.9)	97 (22.9)	77 (18.2)	44 (10.4)	24 (5.7)	17 (4.0)	2.33 ± 1.43
(11) Nurses fear adverse consequences from reporting medication errors	144 (34.0)	79 (18.6)	67 (15.8)	56 (13.2)	51 (12.0)	27 (6.4)	2.70 ± 1.63
(12) The response by the nursing administration does not match the severity of the error	133 (31.4)	75 (17.7)	71 (16.7)	67 (15.8)	52 (12.3)	26 (6.1)	2.78 ± 1.61
(13) Nurses could be blamed if something happens to the patient as a result of the medication error	70 (16.5)	47 (11.1)	62 (14.6)	83 (19.6)	88 (20.8)	74 (17.5)	3.69 ± 1.70
(14) No positive feedback is given for passing medications correctly	103 (24.3)	71 (16.7)	103 (24.3)	75 (17.7)	47 (11.1)	25 (5.9)	2.92 ± 1.51
(15) Too much emphasis is placed on med errors as a measure of the quality of nursing care provided	97 (22.9)	76 (17.9)	90 (21.2)	85 (20.0)	49 (11.6)	27 (6.4)	2.99 ± 1.53
(16) When med errors occur, nursing administration focuses on the individual rather than looking at the systems as a potential cause of the error	54 (12.7)	50 (11.8)	65 (15.3)	91 (21.5)	86 (20.3)	78 (18.4)	3.80 ± 1.64

^*∗*^Min (1); max (6).

**Table 4:** The percentage of perceived medication administration errors reported for each type by the critical care nurses in tertiary hospitals, Kelantan (*n* = 424).

Type of medication errors	Percentage of each type of medication error reported (%)^∗^	
	IV-related MAEs	Non-IV-related MAEs
(1) Wrong route of administration	16.1	18.6
(2) Wrong time of administration	16.5	18.9
(3) Wrong patient	15.4	16.5
(4) Wrong dose	16.3	18.2
(5) Wrong drug	15.6	16.7
(6) Medication is omitted	24.2	26.5
(7) Medication is given but has not been ordered by the physician	15.0	14.7
(8) Medication is administered after the order to discontinue has been written	15.6	15.2
(9) Given to patients with a known allergy	15.3	15.1
(10) Wrong fluid	14.9	
(11) Wrong rate of administration	15.5	

^*∗*^Min (0); max (100).

## Data Availability

The data are not publicly available due to privacy and confidentiality. However, restrictions apply to the availability of hospital data, which are available from the authors with the organization's permission.
